# Sugar-sweetened beverage consumption and metabolic dysfunction-associated steatotic liver disease: a beverage type-specific analysis using Korea National Health and Nutrition Examination Survey

**DOI:** 10.4178/epih.e2025038

**Published:** 2025-07-21

**Authors:** Hong Jae Jeon, Woo Sun Rou, Seok Hyun Kim, Byung Seok Lee, Ha Neul Kim, Hei-Gwon Choi, Jaehee Seo, Hyuk Soo Eun, Sukyoung Jung

**Affiliations:** 1 Department of Internal Medicine, Chungnam National University Sejong Hospital, Sejong, Korea; 2 Department of Internal Medicine, Chungnam National University School of Medicine, Daejeon, Korea; 3 Department of Internal Medicine, Chungnam National University Hospital, Daejeon, Korea; 4 Department of Medical Science, Chungnam National University, Daejeon, Korea; 5 Public Health Policy Office, Chungnam National University Sejong Hospital, Sejong, Korea; 6 Department of Healthcare Policy Research, Korea Institute for Health and Social Affairs, Sejong, Korea

**Keywords:** Beverages, Sweetening agents, Korean traditional drinks, Metabolic dysfunction-associated steatotic liver disease, Korea

## Abstract

**OBJECTIVES:**

Metabolic dysfunction-associated steatotic liver disease (MASLD) is the most prevalent liver disease. Evidence indicates a relationship between sugar-sweetened beverage (SSB) consumption and MASLD development; however, the effects of specific SSB types on MASLD remain underexplored. This study investigated the association between consumption of 3 distinct types of SSBs and MASLD in Korean adults.

**METHODS:**

We analyzed data from the Korea National Health and Nutrition Examination Survey 2012-2016, including 8,310 participants aged 40-64 years. SSB consumption (soft drinks, fruit drinks, and Korean traditional drinks) was assessed using a food frequency questionnaire. MASLD was defined as a hepatic steatosis index ≥36 in the presence of any cardiometabolic risk factor. Logistic regression models were used to estimate odds ratios (ORs) with 95% confidence intervals (CIs), adjusting for relevant covariates.

**RESULTS:**

Compared to non-drinkers, consumption of soft drinks (≥3 serving/wk) was associated with higher odds of MASLD (OR, 1.36; 95% CI, 1.02 to 1.81; p for trend=0.03), while consumption of Korean traditional drinks (≥3 serving/wk) was associated with lower odds of MASLD (OR, 0.63; 95% CI, 0.45 to 0.89; p for trend=0.01). No association was found between fruit drink consumption and MASLD. In sex-stratified analysis (p-interaction=0.38), an inverse association between Korean traditional drinks and MASLD was observed in male (OR, 0.57; 95% CI, 0.34 to 0.95; p for trend=0.03), but not in female (OR, 0.72; 95% CI, 0.45 to 1.14; p for trend=0.17).

**CONCLUSIONS:**

Consuming soft drinks at least 3 times per week was positively associated with MASLD, whereas an equivalent intake of Korean traditional drinks was inversely associated with MASLD.

## GRAPHICAL ABSTRACT

[Fig f2-epih-47-e2025038]

## Key Message

Among 8,310 Korean adults aged 40-64 years, soft drink consumption (≥3 servings/week) increased the odds of MASLD, while the same intake of Korean traditional drinks decreased the odds. These findings suggest that beverage type may be an important dietary factor associated with MASLD.Given the differential associations observed, our findings may help inform dietary recommendations that enable individuals to make more selective beverage choices for better liver health.

## INTRODUCTION

Non-alcoholic fatty liver disease (NAFLD) is one of the most common causes of chronic liver disease, characterized by intrahepatic fat accumulation associated with insulin resistance. The prevalence of NAFLD has risen rapidly in tandem with increasing rates of obesity and diabetes worldwide; in Korea, its prevalence is estimated at 20-30% [1,2]. NAFLD encompasses a spectrum of conditions, ranging from simple steatosis to non-alcoholic steatohepatitis, cirrhosis, and hepatocellular carcinoma. Recently, a multisociety Delphi consensus statement on liver disease nomenclature introduced the concept of steatotic liver disease, encompassing steatosis from various etiologies. Specifically, the term metabolic dysfunction-associated steatotic liver disease (MASLD) is replacing NAFLD, and refers to patients with hepatic steatosis who meet any of 5 cardiometabolic criteria [3,4].

Although MASLD has traditionally been linked to sedentary lifestyles and overnutrition, recent evidence suggests that diets high in sugar may increase the risk of MASLD. Sugar-sweetened beverages (SSBs) are a leading source of dietary sugar worldwide. SSBs contribute to weight gain by providing additional liquid calories and induce hyperinsulinemia through rapid glucose absorption; they also promote the development of cardiometabolic diseases by adversely affecting glycemic control. Fructose from sucrose or high-fructose corn syrup in SSBs bypasses the glycolytic rate-limiting step catalyzed by phosphofructokinase, thereby increasing the substrate available for de novo lipogenesis compared to glucose. This unique hepatic metabolism of fructose is considered a key mechanism underlying MASLD development [5,6].

The average consumption of SSB per capita by Koreans increased by approximately 200% from 2007 to 2020, reflecting a sharp increase compared with the 6% increase in the global average consumption per capita of SSB during a similar period [7,8]. As SSB consumption has surged in Korea, epidemiological studies have linked SSB intake to increased risk of obesity and metabolic syndrome in Korean adults [9], hypertension (particularly among obese participants) [10], and cardiovascular disease among Korean male who do not engage in regular physical activity [11]. These findings highlight the metabolic consequences of SSB consumption. However, emerging data suggest that not all SSBs have the same impact on cardiometabolic health. Several studies indicate that the effects of SSBs on the development of metabolic syndrome [12], type 2 diabetes mellitus [13,14], and cardiovascular disease [15] vary by SSB type, particularly regarding their main ingredients (e.g., soft drinks, fruit drinks, etc.).

Despite this, no study has evaluated the association between SSB consumption and MASLD, specifically examining different beverage types in Korea or globally. Therefore, our aim was to determine whether consumption of soft drinks, fruit drinks, and Korean traditional drinks is associated with the odds of MASLD among Korean adults, using data from the Korea National Health and Nutrition Examination Survey (KNHANES) 2012-2016.

## MATERIALS AND METHODS

### Study population

The KNHANES is an ongoing, cross-sectional, and nationally representative survey conducted by the Korea Disease Control and Prevention Agency to monitor the health and nutritional status of the non-institutionalized Korean population. KNHANES employs a complex multistage probability sampling design to select a representative sample. Data collection includes health interviews, physical examinations, and nutrition surveys. Further details regarding KNHANES are available online (https://knhanes.kdca.go.kr/knhanes/main.do).

Of the 10,240 adults aged 40-64 years who completed the food frequency questionnaire (FFQ)—an age range chosen to minimize diagnostic uncertainty of MASLD in younger individuals—we excluded participants with implausible energy intakes (<1st or >99th percentile: <700 or >4,500 kcal; n=188), those who were pregnant or lactating (n=9), individuals with self-reported severe diseases (e.g., heart disease, cancer, liver cirrhosis; n=636), and participants missing information on MASLD assessment (n=971) or socio-demographic variables (n=126). The final analytic sample comprised 8,310 adults (3,240 male and 5,070 female; Supplementary Material 1).

### Assessment of sugar-sweetened beverages consumption

Trained dieticians collected dietary data using a validated, dish-based, semi-quantitative FFQ to assess the intake of 112 food and dish items [16]. Participants reported both the frequency and average portion sizes of these 112 items over the previous year. The FFQ provided 9 frequency categories (never or <1/mo, once/mo, 2-3 times/mo, once/wk, 2-4 times/wk, 5-6 times/wk, once/day, twice/day, and 3 times/day) and 3 portion sizes (0.5, 1.0, or 1.5-2.0 times the standard serving). The main exposures were the consumption of soft drinks (e.g., cola, cider, carbonated fruit drinks), commercial fruit drinks (including 100% fruit juice, mixed fruit and vegetable juice, tomato juice, and fruit-flavored drinks, but excluding homemade juice), and Korean traditional beverages (e.g., roasted grain powders and rice-based beverages such as Sikhye). One serving was defined as 200 mL for each beverage type. SSB consumption (servings/wk) was categorized into 3 groups for analysis based on the distribution of intake for each beverage. The 95th percentile values were 3 servings/wk for soft drinks and fruit drinks, and 1.5 servings/wk for Korean traditional drinks. To maintain consistency, participants were classified as non-drinkers, drinkers of less than 3 servings/wk, and drinkers of 3 or more servings/wk.

### Assessment of metabolic dysfunction-associated steatotic liver disease

MASLD was defined as a hepatic steatosis index (HSI) ≥36 in the presence of at least 1 of 5 cardiometabolic risk factors.

#### Definition of steatotic liver disease using HSI

During health examinations, trained staff measured anthropometrics according to standardized procedures and using calibrated equipment [17]. Standing height (cm) was measured using a stadiometer, and body weight (kg) was measured with participants in light clothing using a metric scale. Body mass index (BMI) was calculated as weight (kg) divided by height squared (m²). Waist circumference (WC) was measured at the midpoint between the lower border of the rib cage and the iliac crest using an inelastic tape. Laboratory tests included serum aspartate aminotransferase (AST, IU/L), alanine aminotransferase (ALT, IU/L), fasting plasma glucose (mg/dL), glycated hemoglobin (HbA1c, %), high-density lipoprotein (HDL)-cholesterol (mg/dL), and triglycerides (mg/dL). Blood samples were drawn after participants had fasted for at least 8 hours. AST and ALT were measured using a Hitachi 7600-210 automatic analyzer (Hitachi, Tokyo, Japan).

Diabetes was defined as any of the following: (1) an affirmative response to “Other than during pregnancy, have you ever been told by a doctor or another health professional that you have diabetes or sugar diabetes?”; (2) current use of diabetes medications, including insulin; or (3) fasting plasma glucose ≥126 mg/dL [18].

The presence of steatotic liver disease was assessed using the HSI using the following formula [19]:


$$\text{HSI}=8\times \frac{\text{ALT}}{\text{AST}}+BMI(+2,if\;diabetes;+2,if\;female)$$

The median HSI value in this study was 32.4 (range, 19.5 to 66.1). The presence of steatotic liver disease was defined as a total HSI score ≥36.

#### Definition of cardiometabolic risk

Cardiometabolic risk was defined as at least 1 of the following: (1) BMI ≥23 kg/m^2^ or WC ≥90 cm for male and ≥85 cm for female; (2) fasting plasma glucose ≥100 mg/dL, HbA1C ≥5.7%, physician-diagnosed type 2 diabetes, or current administration of medication for type 2 diabetes; (3) systolic blood pressure ≥130 mmHg or diastolic blood pressure ≥85 mmHg or current administration of antihypertensive medication; (4) plasma triglycerides ≥150 mg/dL or current administration of lipid-lowering medication; (5) plasma HDL-cholesterol ≤40 mg/dL for male and ≤50 mg/dL for female or current administration of lipid-lowering medication [20].

### Assessment of covariates

Covariates included age (years), sex (male or female), residential area (urban or rural), education level (less than high school graduate or high school graduate or higher), monthly household income (quartiles of equivalized household income), marital status (married or not), current smoking status (yes or no), current drinking status (yes or no), physical activity (metabolic equivalent of task [MET]-min/wk] [21], total energy intake (kcal/day), and healthy eating index (HEI; score range, 0 to 100) [22].

### Statistical analysis

We summarized the general characteristics of study participants using weighted means and standard errors (SEs) for continuous variables, and weighted prevalence and SEs for categorical variables, stratified by SSB consumption group. Differences between groups were evaluated using analysis of variance for continuous variables and the Rao-Scott chi-square test for categorical variables.

Logistic regression models were used to estimate covariate-adjusted odds ratios (ORs) and 95% confidence intervals (CIs) for MASLD, comparing participants consuming less than 3 servings per week and those consuming 3 or more servings per week with non-drinkers, for each SSB category. Two multivariable-adjusted models were presented: Model 1 adjusted for age, sex, residential area, education, income, marital status, current smoking, current drinking, physical activity, and total energy intake; Model 2 further adjusted for HEI. To assess linear trends, the median SSB consumption in each category was modeled as a continuous variable in the logistic regression. Stratified analyses by sex were conducted to further examine associations between SSB consumption and MASLD. Additional stratified analyses were performed according to age, residential area, education, income, marital status, smoking, drinking, MET-min/wk, total energy intake, and HEI.

The data were analyzed using PROC SURVEY procedures in SAS version 9.4 (SAS Institute Inc., Cary, NC, USA), with survey weights applied to account for KNHANES’s complex sampling design. All tests were 2-sided, with statistical significance set at p-value<0.05.

### Ethics statement

The KNHANES study protocols were approved by the KDCA Ethics Review Board. All participants provided written informed consent before participation, and all survey data were anonymized before analysis. Our study was deemed exempt by the Chungnam National University Hospital Institutional Review Board (2023-08-091) because we used only publicly available data without identifiable information.

## RESULTS

### Participants’ characteristics

Table 1 presents the general characteristics of participants according to their SSB consumption. Participants who consumed 3 or more servings of soft drinks per week were more likely to be younger, male, more educated, have higher income, be current smokers and current drinkers, and be unmarried. They also had higher total energy intake and fat intake, lower HEI scores, and were less physically active. Participants who consumed 3 or more servings of fruit drinks per week also tended to be younger, male, more educated, have higher income, be current smokers and current drinkers, and had higher total energy and fat intake, but were less physically active. In contrast, participants who consumed 3 or more servings of Korean traditional drinks per week were more likely to be male, current smokers, married, reside in rural areas, and have higher total energy intake, fat intake, and HEI scores.

### Associations between sugar-sweetened beverage consumption and the prevalence of metabolic dysfunction-associated steatotic liver disease

Among the total study population, the prevalence of HSI-defined MASLD was 25.3%. Stratified by age group, the prevalence was 25.3% among participants aged 40-49 years, 25.1% among those aged 50-59 years, and 26.1% among those aged 60-69 years (data not shown). Table 2 summarizes the associations between SSB consumption and the presence of HSI-defined MASLD. In the multivariable-adjusted model, participants who consumed soft drinks ≥3 servings/wk had 1.4 times higher odds of MASLD than non-drinkers (OR, 1.37; 95% CI, 1.03 to 1.82; p for trend=0.019), while participants who consumed Korean traditional drinks ≥3 servings/wk had approximately 0.7 times lower odds of MASLD compared to non-drinkers (OR, 0.66; 95% CI, 0.47 to 0.93; p for trend=0.015). These associations remained after additional adjustment for HEI (OR, 1.36; 95% CI, 1.02 to 1.81; p for trend=0.025 for soft drinks; OR 0.63; 95% CI, 0.45 to 0.89; p for trend=0.008 for Korean traditional drinks). No significant association was observed between fruit drink consumption and MASLD.

### Stratified analyses

**Figure 1.**
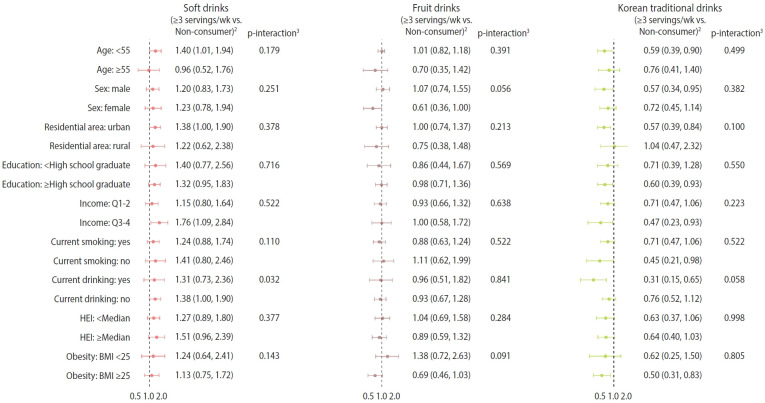
Stratified associations between specific types of beverage consumption^1^ and the presence of hepatic steatosis index-defined metabolic dysfunction associated steatotic liver disease, Korea National Health and Nutrition Examination Survey 2012-2016. Values are presented as odds ratio (95% confidence interval). HEI, healthy eating index; BMI, body mass index; METs, metabolic equivalent of task. ^1^One serving is defined as 200 mL. ^2^Odds ratios and 95% CIs were obtained using the logistic regression model after adjusting for age, sex, residential area, education level, monthly household income level, marital status, current smoking, current drinking, METs/wk, total energy intake, and healthy eating index. ^3^p-values for interactions were determined by including the cross-product term of the each beverage consumption and covariates.

Figure 1 displays the associations between SSB consumption and MASLD across various subgroups. In the multivariable-adjusted model, no significant differences were observed in the association of soft drink and fruit drink consumption with MASLD between male and female. Although there was no significant association among female (OR, 0.72; 95% CI, 0.45 to 1.14; p for trend=0.17), male who consumed Korean traditional drinks ≥3 servings/wk had significantly lower odds of MASLD compared to non-drinkers (OR, 0.57; 95% CI, 0.34 to 0.95; p for trend=0.03). For soft drinks, a significant interaction effect was found by alcohol drinking status (p for interaction=0.032), but the estimates were similar for current drinkers and non-drinkers (OR, 1.31 vs. 1.38). No significant interaction effects were found for other covariate subgroups, including HEI levels.

## DISCUSSION

In this cross-sectional study of Korean adults, we found that higher consumption of soft drinks—specifically, ≥3 servings/wk—was significantly associated with an increased risk of MASLD, while greater consumption of traditional Korean drinks (≥3 servings/wk) was significantly associated with a lower risk of MASLD, after adjustment for multiple covariates. No association was observed between fruit drink consumption and MASLD. When stratified by sex, a significant inverse association between Korean traditional drinks and MASLD was identified in male, but not in female. Additionally, stratification by alcohol consumption revealed that the association between soft drink consumption and MASLD was significant only among non-drinkers, but not among alcohol consumers.

Globally, SSB consumption is rising, particularly in low-income and middle-income countries, and already exceeds recommended daily free sugar intake in many high-income countries [6,8]. Several meta-analyses and prospective cohort studies provide strong evidence linking SSB consumption to weight gain and cardiometabolic diseases. Since initial Israeli studies reported that patients with NAFLD consumed more SSBs—especially soft drinks—than the general population, accumulating evidence has pointed to a relationship between SSB intake and NAFLD development [6,23,24].

To our knowledge, this is the first study to examine the effects of SSB consumption on MASLD using the newly proposed concept. Consistent with our findings, a cross-sectional study of United States adults from the Framingham Heart Study’s Offspring and Third Generation cohorts reported a 56% higher risk of NAFLD among daily SSB consumers (≥8 ounces/day), as defined by abdominal scan, compared to non-consumers [25]. Another cross-sectional study of United States adults from National Health and Nutrition Examination Survey showed that individuals who consumed more than 1 serving/day (>8 ounces/day) had 1.5 times higher odds of liver stiffness (>7 kPa) and 2.1 times higher odds of hepatic steatosis (controlled attenuation parameter >248) than non-SSB consumers [26]. An Australian prospective cohort study found that SSB consumption >197.2 g/day was associated with an increased risk of NAFLD [27]. In China, a cross-sectional study found that soft drink consumption exceeding 1 cup (200-230 mL) per week was associated with a higher prevalence of NAFLD, defined by elevated ALT (OR, 1.32; 95% CI, 1.13 to 1.53), and a prospective cohort study demonstrated that the same amount of SSB consumption was linked to increased NAFLD incidence (hazard ratio, 1.18; 95% CI, 1.03 to 1.34) [28,29]. Despite similarities in the direction of association between the current and previous studies, differences exist in the definitions of liver disease and the amount of SSB consumption assessed between Asian and Western countries.

In the present study, unlike soft and Korean traditional drinks, fruit drinks were not associated with MASLD. Although fruits contain fructose, they are generally considered to have low obesogenic potential and may be beneficial in the prevention and progression of NAFLD [30,31]. This may be due to the presence of antioxidants in fruits, which can counteract the metabolic effects of fructose [32]. Similarly, several studies have suggested that 100% fruit juice may help prevent and ameliorate NAFLD, possibly due to its phytochemical content, such as anthocyanins, polyphenols, and resveratrol [33]. For example, a cross-sectional study of European adults reported a beneficial association between moderate fruit juice intake (≤2 servings/wk) and NAFLD (prevalence ratio, 0.92; 95% CI, 0.88 to 0.97) [34]. However, another cross-sectional study of Dutch adults found that individuals in the highest tertile of energy-adjusted fructose intake from fruit juice had 1.04-fold higher intrahepatic lipid levels compared to those in the lowest tertile [35]. In our study, the lack of association between fruit drink consumption and MASLD may be explained by the fact that our definition of fruit drinks included not only 100% fruit juice but also fruit-flavored drinks and sugar-added fruit juices. As fruit drinks were assessed using a single aggregated item, distinguishing specific fruit drink types was not possible in the current analysis. Future research should classify fruit drinks by type to better evaluate the effects of fruit juice consumption on MASLD.

Interestingly, we observed that consumption of Korean traditional drinks, such as Sikhye and Misu, was inversely associated with MASLD risk. While mass-produced Korean traditional drinks (ultra-processed) may contain sugar levels comparable to soft drinks, those prepared at home using traditional methods may contain less added sugar. Unfortunately, we could not differentiate between industrially produced and homemade Korean traditional drinks, as they were assessed as a single item in the FFQ. This limitation requires further investigation. Additionally, malt extracts used in the preparation of Sikhye have been reported to possess antioxidant activity, which may help prevent NAFLD [36,37]. Several studies have shown that hepatic fat content decreases when SSBs are replaced with low-calorie sweetened (LCS) beverages [38-40]. However, the long-term effects of LCS beverage consumption on cardiometabolic health remain unclear. Therefore, many diabetes and nutrition societies do not endorse LCS beverages and instead recommend water over SSBs. Nonetheless, water may not be a feasible alternative for individuals who habitually consume soft drinks. In such cases, non-mass-produced Korean traditional drinks with lower sugar content may serve as a practical substitute for soft drinks to help reduce MASLD risk. Randomized controlled trials are needed to determine whether replacing soft drinks with Korean traditional drinks can effectively prevent MASLD.

In our sex-stratified analyses, an increased risk of MASLD was observed with higher soft drink consumption in female, but not in male. Generally, the overall prevalence of MASLD is higher in male than in female, and evidence suggests that estrogen exerts protective effects against MASLD. However, some studies have indicated that female may be more susceptible to fructose-induced fatty liver disease. For example, in an experimental study of Wistar rats (n=24 male and 24 female) fed a high-fructose diet for 10 weeks, Hyer et al. [41] reported that female rats exhibited extensive NAFLD-like liver pathology, whereas male rats displayed minimal hepatic changes. In a randomized crossover study of 16 healthy adults (8 male and 8 female), Low et al. [42] found that intake of a high-fructose meal led to a greater increase in postprandial hepatic de novo lipogenesis in female than in male. However, previous experimental studies in mice fed altered diets have reported conflicting results, sometimes finding more severe disease in male; thus, further research is needed to clarify sex-specific effects of fructose on NAFLD [43,44].

This study has several limitations. First, possible misclassification of MASLD may have occurred because MASLD was defined using the HSI scoring system rather than liver biopsy. Nevertheless, the HSI is a screening tool specifically developed from Korean health check-up data and is well validated in Korean and Asian populations, so the diagnostic accuracy for NAFLD in this Korean sample is expected to be high [19,45,46]. Second, inevitable measurement errors inherent to the FFQ may have led to misclassification of SSB consumption, potentially attenuating the observed associations. However, dietary intake assessed by FFQ is cost-effective in large samples and accurately reflects usual intake. Third, fruit drinks and Korean traditional drinks were each assessed as a single item, making it impossible to distinguish between potentially beneficial and harmful types (e.g., 100% fruit juice without added sugar, homemade traditional drinks with lower sugar content). This limitation may influence the direction or strength of observed associations. Further studies with more detailed information on SSB types are needed to address this issue. Fourth, the cross-sectional design of this study limits the ability to infer causality between SSB consumption and MASLD. Long-term prospective studies are necessary to evaluate the causal relationship. Fifth, the magnitude of the associations may have been affected by residual confounding from unmeasured or unknown factors. Finally, our study population consisted of Korean adults aged 40-64 years, so the results may not be generalizable to other populations or age groups. The prevalence of MASLD among participants in our study was 25.3%, which is similar to previously reported NAFLD prevalence in Korean adults [1]. In the broader KNHANES population, the prevalence of MASLD was 18.2% among those in their 20s and unexpectedly higher at 25.6% in those in their 30s. These findings highlight the need for further research including not only middle-aged but also younger populations.

In conclusion, consumption of soft drinks at least 3 times/wk (600 mL or more) was positively associated with MASLD occurrence, whereas consuming an equivalent amount of Korean traditional drinks was inversely associated with MASLD in this nationally representative sample of Korean adults. Our findings suggest that the effect of SSBs on MASLD varies by beverage type; soft drink consumption should be limited, while moderate consumption of Korean traditional drinks may be beneficial for liver health in Korean adults.

## Figures and Tables

**Figure f2-epih-47-e2025038:**
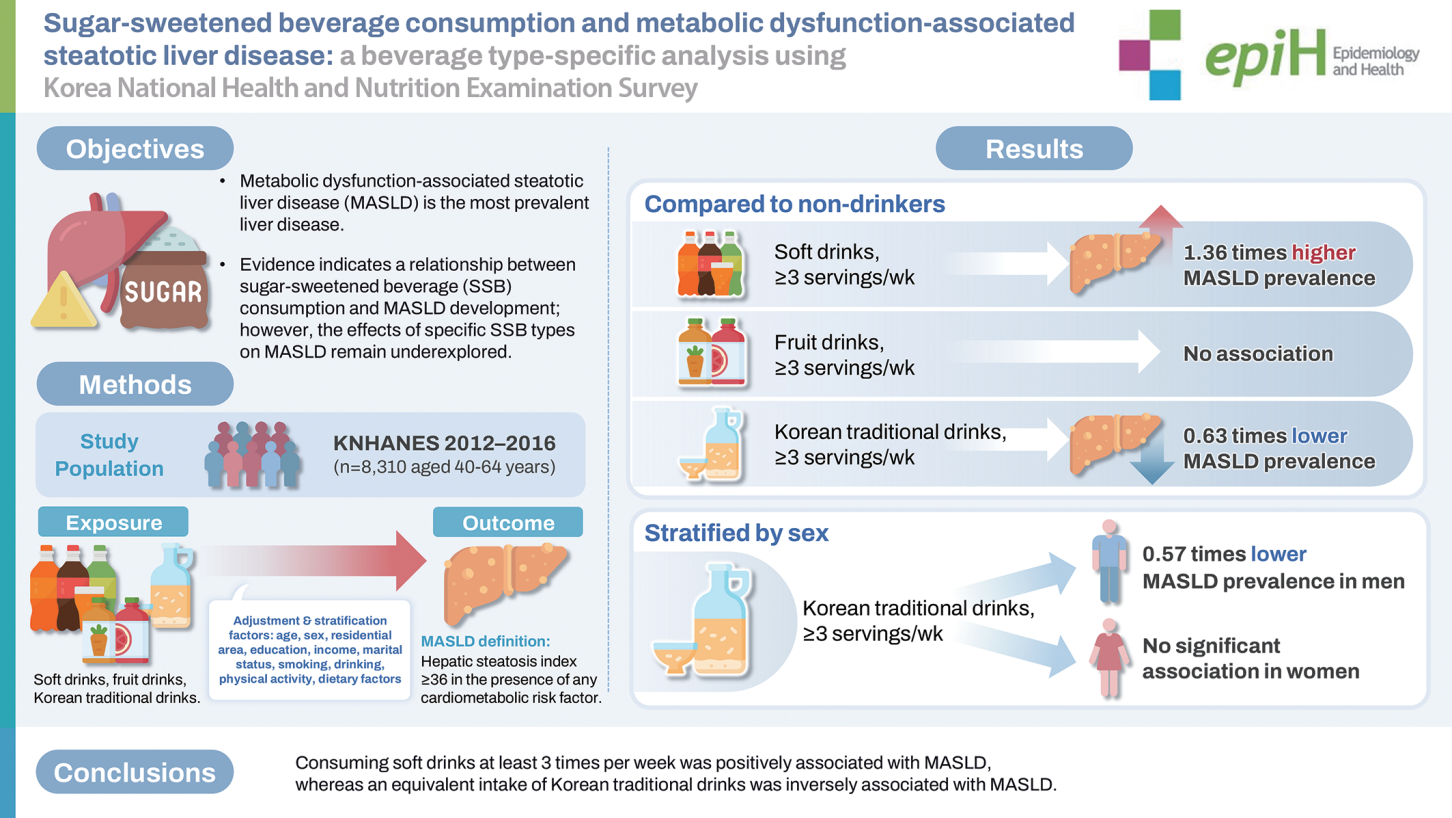


**Table 1.** Demographic and lifestyle characteristics of study participants according to sugar-sweetened beverage consumption, KNHANES 2012-2016 (n=8,310)

Characteristics	Soft drinks (servings/wk)^1^				Fruit drinks (servings/wk)^1^				Korean traditional drinks (servings/wk)			
	Non-drinker	0-3	≥3	p-value^2^	Non-drinker	0-3	≥3	p-value^2^	Non-drinker	0-3	≥3	p-value^2^
Median (min, max) SSB intake	0 (0, 0)	0.5 (0.1, 2.8)	3.0 (3.0, 21.0)		0 (0, 0)	0.5 (0.1, 2.8)	3.0 (3.0, 14.0)		0 (0, 0)	0.3 (0.1, 1.5)	3.8 (3.0, 21.0)	
Sample size (n)	5,621	2,240	449		5,390	2,445	475		5,569	2,398	343	
Demographic												
Age (yr)	51.8 (0.1)	48.5 (0.2)	47.3 (0.3)	<0.001	51.4 (0.1)	49.2 (0.2)	48.6 (0.4)	<0.001	50.6 (0.1)	50.4 (0.2)	50.2 (0.4)	0.258
Sex												
Male	43.6 (0.7)	56.4 (1.1)	73.3 (2.1)	<0.001	43.9 (0.7)	56.9 (1.0)	64.5 (2.4)	<0.001	48.9 (0.7)	48.7 (1.1)	58.6 (2.8)	0.006
Female	56.4 (0.7)	43.6 (1.1)	26.7 (2.1)		56.1 (0.7)	43.1 (1.0)	35.5 (2.4)		51.1 (0.7)	51.3 (1.1)	41.4 (2.8)	
Residential area												
Urban	84.2 (1.2)	82.0 (1.5)	81.6 (2.5)	0.094	83.9 (1.3)	82.7 (1.5)	82.5 (2.3)	0.471	84.4 (1.2)	81.5 (1.5)	81.4 (2.9)	0.026
Rural	15.8 (1.2)	18.0 (1.5)	18.4 (2.5)		16.1 (1.3)	17.3 (1.5)	17.5 (2.3)		15.6 (1.2)	18.5 (1.5)	18.6 (2.9)	
Socioeconomic												
Education level												
<High school	29.0 (0.8)	19.3 (1.0)	16.4 (2.0)	<0.001	29.2 (0.8)	19.0 (0.9)	19.0 (2.1)	<0.001	25.3 (0.8)	25.3 (1.1)	25.7 (2.7)	0.990
≥High school	71.0 (0.8)	80.7 (1.0)	83.6 (2.0)		70.8 (0.8)	81.0 (0.9)	81.0 (2.1)		74.7 (0.8)	74.7 (1.1)	74.3 (2.7)	
Household income												
Q1	9.5 (0.5)	6.9 (0.6)	8.9 (1.4)	0.012	10.4 (0.6)	5.9 (0.5)	5.6 (1.2)	<0.001	9.3 (0.5)	7.7 (0.6)	6.5 (1.5)	0.253
Q2	23.6 (0.8)	22.2 (1.1)	26.8 (2.5)		23.9 (0.8)	22.6 (1.1)	22.4 (2.2)		23.8 (0.8)	22.7 (1.1)	21.3 (2.9)	
Q3	29.8 (0.9)	32.6 (1.2)	30.3 (2.6)		29.7 (0.9)	33.5 (1.2)	26.5 (2.5)		30.1 (0.9)	31.7 (1.2)	32.6 (3.1)	
Q4	37.1 (1.0)	38.3 (1.5)	34.1 (2.7)		36.0 (1.1)	38.0 (1.4)	45.5 (2.8)		36.9 (1.1)	37.9 (1.4)	39.6 (3.5)	
Marital status												
Not married	3.0 (0.3)	4.4 (0.6)	5.2 (1.3)	0.023	3.5 (0.3)	4.0 (0.5)	2.4 (0.8)	0.324	4.1 (0.4)	2.6 (0.4)	1.3 (0.7)	0.003
Married	97.0 (0.3)	95.6 (0.6)	94.8 (1.3)		96.5 (0.3)	96.0 (0.5)	97.6 (0.8)		95.9 (0.4)	97.4 (0.4)	98.7 (0.7)	
Lifestyle												
Current smoking												
No	80.5 (0.7)	75.4 (1.2)	61.6 (2.6)	<0.001	79.1 (0.7)	76.3 (1.1)	71.3 (2.4)	0.001	77.2 (0.7)	79.8 (1.0)	72.0 (3.1)	0.017
Yes	19.5 (0.7)	24.6 (1.2)	38.4 (2.6)		20.9 (0.7)	23.7 (1.1)	28.7 (2.4)		22.8 (0.7)	20.2 (1.0)	28.0 (3.1)	
Current drinking												
No	25.1 (0.7)	19.3 (0.9)	21.7 (2.2)	<0.001	25.2 (0.7)	20.0 (0.9)	18.2 (2.1)	<0.001	22.7 (0.7)	24.0 (1.0)	25.1 (2.7)	0.427
Yes	74.9 (0.7)	80.7 (0.9)	78.3 (2.2)		74.8 (0.7)	80.0 (0.9)	81.8 (2.1)		77.3 (0.7)	76.0 (1.0)	74.9 (2.7)	
MET-min/wk^3^	2,488 (66)	2,229 (73)	2,407 (203)	0.030	2,494 (69)	2,234 (64)	2,448 (163)	0.017	2,385 (59)	2,390 (77)	2,993 (257)	0.068
Diet												
Energy (kcal/day)	1,915 (10)	2,057 (17)	2,333 (38)	<0.001	1,900 (11)	2,064 (15)	2,395 (36)	<0.001	1,893 (10)	2,143 (16)	2,272 (52)	<0.001
Carbohydrate (%kcal/day)	70.5 (0.1)	69.3 (0.2)	68.0 (0.4)	<0.001	70.5 (0.1)	69.4 (0.2)	67.5 (0.4)	<0.001	70.3 (0.1)	69.4 (0.2)	70.0 (0.4)	<0.001
Protein (%kcal/day)	13.4 (0.04)	13.5 (0.05)	13.7 (0.13)	0.049	13.3 (0.04)	13.6 (0.05)	14.0 (0.12)	<0.001	13.3 (0.04)	13.6 (0.05)	13.3 (0.13)	<0.001
Fat (%kcal/day)	16.2 (0.1)	17.2 (0.1)	18.4 (0.3)	<0.001	16.2 (0.1)	17.1 (0.1)	18.5 (0.3)	<0.001	16.4 (0.1)	17.0 (0.1)	16.7 (0.3)	<0.001
HEI	67.8 (0.2)	67.0 (0.3)	64.8 (0.7)	<0.001	67.2 (0.2)	67.6 (0.3)	68.7 (0.7)	0.061	66.8 (0.2)	68.3 (0.3)	69.9 (0.7)	<0.001

Values are weighted and presented as mean (SE) for continuous variables and percentage (SE) for categorical variables. KNHANES, Korea National Health and Nutrition Examination Survey; SSB, sugar-sweetenedbeverage; METs, metabolic equivalent of task; HEI, healthy eating index; SE, standard error.

1 One serving is defined as 200 mL.

2 p-values for differences by sugar-sweetened beverage intake categories were obtained using general linear models for continuous variables and Rao-Scott chi-square tests for categorical variables.

3 The MET-min/wk was quantified by multiplying the frequency, duration, and intensity of any physical activity engaged in during a typical week. Vigorous or moderate activities were assigned 8.0 and 4.0 MET values, respectively.

**Table 2.** Associations between specific types of beverage consumption and the presence of hepatic steatosis index-defined metabolic dysfunction associated steatotic liver disease, KNHANES 2012-2016^1^

Variables	Consumption categories (servings/wk)^2^			
	Non-drinker	0-3	≥3	p for trend^3^
Soft drinks				
No. of participants	5,621	2,240	449	
No. of cases (%)	1,280 (23.2)	625 (28.5)	143 (31.5)	
Multivariable-adjusted 1^4^	1.00 (reference)	1.27 (1.10, 1.48)	1.37 (1.03, 1.82)	0.019
Multivariable-adjusted 2^5^	1.00 (reference)	1.25 (1.08, 1.46)	1.36 (1.02, 1.81)	0.025
Fruit drinks				
No. of participants	5,390	2,445	475	
No. of cases (%)	1,323 (24.8)	616 (26.2)	109 (26.0)	
Multivariable-adjusted 1^4^	1.00 (reference)	1.01 (0.88, 1.16)	0.92 (0.70, 1.23)	0.608
Multivariable-adjusted 2^5^	1.00 (reference)	1.02 (0.88, 1.17)	0.95 (0.72, 1.27)	0.761
Korean traditional drinks				
No. of participants	5,569	2,398	343	
No. of cases (%)	1,406 (25.9)	569 (24.3)	73 (22.2)	
Multivariable-adjusted 1^4^	1.00 (reference)	0.87 (0.75, 1.01)	0.66 (0.47, 0.93)	0.015
Multivariable-adjusted 2^5^	1.00 (reference)	0.87 (0.75, 1.02)	0.63 (0.45, 0.89)	0.008

Values are presented as odds ratio (95% confidence interval). KNHANES, Korea National Health and Nutrition Examination Survey; METs, metabolic equivalent of task.

1 Logistic regression models were used to estimate odds ratios and their corresponding 95% confidence intervals for the presence of metabolic dysfunction associated steatotic liver disease.

2 One serving is defined as 200 mL.

3 p-for trends was determined by treating the median value of each type of beverage consumption as a continuous variable using the logistic regression model.

4 Results were from the logistic regression model after adjusting for age, sex, residential area, education level, monthly household income level, marital status, current smoking, current drinking, METs/wk, and total energy intake.

5 Results were from the logistic regression model after additionally adjusting for healthy eating index.

## Supplementary materials

Supplementary Material 1. Study participant flow chart, Korea National Health and Nutrition Examination Survey (KNHANES) adults aged 40–64 years, 2012–2016
