# Supplementary material for: Sugar-sweetened beverage consumption and metabolic dysfunction-associated steatotic liver disease: a beverage type-specific analysis using Korea National Health and Nutrition Examination Survey
Source: Epidemiol Health. 2025 Jul 21;47:e2025038. doi: 10.4178/epih.e2025038 (PMC12531465; doi:10.4178/epih.e2025038)
Supplement: Supplementary Material 1. — Study participant flow chart, Korea National Health and Nutrition Examination Survey (KNHANES) adults aged 40–64 years, 2012–2016 [file epih-47-e2025038-Supplementary-1.docx]

**Supplementary material**

**Supplement to:** Sugar-sweetened beverage consumption and metabolic dysfunction associated steatotic liver disease: a beverage type-specific analysis using KNHANES

Participants aged 40–64 years who completed food frequency questionnaire in NHANES 2012–2016 (n=10,240)

Incomplete or implausible intake

(n=188)

Pregnant or lactating women

(n=2)

(n=17,135)

Valid dietary intake

(n=10,052)

Pregnant or lactating women

(n=9)

(n=17,135)

(n=17,135)

Excluding pregnant or lactating women

(n=10,043)

Self-reported severe diseases

(n=636)

(n=17,135)

No self-reported severe diseases

(n=9,407)

Missing information on metabolic dysfunction associated steatotic liver disease assessment (n=971) or covariates (n=126)

Final analytic sample

(n=8,310)

**Supplemental Material 1. Study participant flow chart, Korea National Health and Nutrition Examination Survey (KNHANES) adults aged 40–64 years, 2012–2016**
